# Isolation and Characterization of the Stress-Tolerant *Candida tropicalis* YHJ1 and Evaluation of Its Xylose Reductase for Xylitol Production From Acid Pre-treatment Wastewater

**DOI:** 10.3389/fbioe.2019.00138

**Published:** 2019-07-02

**Authors:** Seonghun Kim, Jinhyuk Lee, Bong Hyun Sung

**Affiliations:** ^1^Jeonbuk Branch Institute, Korea Research Institute of Bioscience and Biotechnology, Jeongeup, South Korea; ^2^Genome Editing Research Center, Korea Research Institute of Bioscience and Biotechnology, Daejeon, South Korea; ^3^Synthetic Biology and Bioengineering Research Center, Korea Research Institute of Bioscience and Biotechnology, Daejeon, South Korea; ^4^Department of Biosystems and Bioengineering, KRIBB School of Biotechnology, University of Science and Technology, Daejeon, South Korea; ^5^Department of Bioinformatics, KRIBB School of Bioscience, University of Science and Technology, Daejeon, South Korea

**Keywords:** *Candida tropicalis* YHJ1, stress tolerance, sequential acid-/alkali-pretreatment, acid pretreatment wastewater, xylose, xylitol fermentation, xylose reductase

## Abstract

A stress-tolerant yeast was isolated from honey using acid hydrolysate generated from sequential acid-/alkali-pretreatment of empty palm fruit bunch fiber (EPFBF). The isolated yeast was identified molecularly, taxonomically, and morphologically as *Candida tropicalis* YHJ1, and analyzed for application in xylitol production. The isolated yeast showed stress tolerance toward various chemical reagents and could grow with up to 600 g/L xylose in the culture medium. This yeast also had a broad carbohydrate utilization spectrum, and its xylitol yield was greatest in medium supplemented with xylose as the sole carbon source. In batch fermentation for xylitol production, the yeast could convert xylose prepared from acidic EPFBF pretreatment wastewater into xylitol. Interestingly, *C. tropicalis* YHJ1 xylose reductase, containing a Ser279 residue, exhibited more effective xylitol conversion compared to orthologous *Candida* enzymes containing Leu279 or Asn279; this improvement was associated with NADPH binding, as predicted through homologous structure modeling and enzyme kinetic analysis. Taken together, these results show a novel stress-tolerant yeast strain that may be applicable to xylitol production from toxic lignocellulosic byproducts.

## Introduction

Lignocellulosic biomass is a renewable resource for production of biofuels and chemical monomers through the biorefinery process (Brethauer and Studer, 2015). Such biomass consists primarily of three polymeric materials, cellulose, hemicellulose, and lignin. These main components interact strongly with each other, and disturbance of their rigid structures is needed to break down the polymeric substances to sugar monomers, oligomers, or other aromatic derivatives (Brethauer and Studer, 2015). Basically, the biorefinery process might allow for recycling of polymeric biomass through a monomerization process to obtain fuels and the building blocks of polymers used for biochemical engineering processes (Silveira et al., 2015; Capolupo and Faraco, 2016). The biorefinery process using lignocellulose involves four main steps: physical and chemical pretreatment of the lignocellulosic biomass, enzymatic hydrolysis of the pretreated biomass, fermentation of the resulting sugars, and finally purification or distillation of the products. Different pretreatment procedures are employed for various types of lignocellulosic biomass, which may influence the enzyme conversion efficiency and productivity of fermentation (Alvira et al., 2010; Silveira et al., 2015;Capolupo and Faraco, 2016).

Chemical pretreatment has generally been considered the conventional approach to enhancing cellulose content per gram of biomass through elimination of lignin and hemicellulose (Silveira et al., 2015; Capolupo and Faraco, 2016). On the other hand, pretreatment processes generate wastewater containing high levels of chemicals in the hemicellulose or lignin fractions. Treatment of this wastewater accounted for approximately 20% of overall production costs (Humbird et al., 2011). Wastewater reuse is necessary for this process to be considered a sustainable or green technology, and the application of alternative processes to reduce non-utilized materials can keep overall production costs low (Capolupo and Faraco, 2016; Kim, 2019).

Among the various different types of pretreatment technologies, sequential acid-/alkali-pretreatment is effective for extracting hemicellulose, as well as for enhancing the cellulose content per unit biomass of empty palm fruit bunch fibers (EPFBFs) (Kim et al., 2012a; Kim and Kim, 2013). Repeated use of the acidic wastewater generated during the pretreatment process allows accumulation of the hydrolyzed hemicellulose fraction from EPFBF, which can be further used to produce xylitol, with hydrolyzed xylose produced in the post-pretreatment solution by an adapted yeast strain (Kim, 2019).

Nevertheless, the hemicellulose hydrolysate in the acidic pretreatment wastewater contains thermally degraded compounds, such as furfural and acetic acetate, as well as other aliphatic and aromatic compounds that are known to be toxic and inhibitory to microbial fermentation (Palmqvist and Hahn-Hägerdal, 2000; Jönsson and Martín, 2016). To overcome these inhibitory compounds and allow reutilization of the highly contented xylose present in the acidic pretreatment solution, a xylitol-fermenting yeast was adapted to the pretreatment solution through repeated batch cultivation (Liu, 2011; Mpabanga et al., 2012; Wang et al., 2015; Kim, 2019). The adapted yeast strain may have evolved tolerance toward the stresses caused by these inhibitory compounds, and thus improved cell growth and xylitol production.

Isolation of a stress-tolerant strain rather than an adapted strain is an alternative approach to obtaining a potential microorganism for converting xylose in acid wastewater to xylitol. In this study, we isolated a stress-tolerant yeast from a honey jar using acidic hemicellulose extracts generated through sequential acid-/alkali-pretreatment of EPFBFs. A strain with resistance against various chemical stress reagents was identified molecularly and taxonomically as *Candida tropicalis* YHJ1 for application in xylitol production. Additionally, xylose reductase (XR), the first-step enzyme in xylose metabolism, was also characterized in the isolated yeast and compared with recombinant mutant enzymes through computational homologous modeling for substrate binding.

## Materials and Methods

### Material

The EPFBF hydrolysate was prepared from the acid extract fraction for pretreatment through the sequential acid-/alkali-pretreatment process (Kim et al., 2012a; Kim, 2019).

### Strain Isolation and Culture Condition

A xylose-utilizing yeast strain was isolated from honey using the soluble sugar in EPFBF acidic hydrolysate solution as the sole carbon source. Honey streaked on the wall of the honey jar was inoculated into minimal medium containing neutralized hydrolysate and 0.67% (w/v) yeast nitrogen base, adjusted to pH 6.0; then, the culture was incubated at 30°C with 240 rpm shaking for 120 h. After the first round of cultivation, 1/100 of the culture volume was inoculated into fresh medium, and then cultivated under the conditions described above for 72 h. This enrichment procedure was repeated five times. After enrichment, diluted culture was spread on an agar plate supplemented with the same medium composition used for the minimal medium, and the plate was then incubated at 30°C. Single colonies were isolated from the plates and cultivated in YPD broth (3 g/L yeast extract, 5 g/L peptone, and 10 g/L glucose). Cells were harvested from the pure culture, resuspended in 30% (v/v) glycerol (v/v), and maintained at −80°C for further strain identification.

### Molecular Identification of the Isolated Strain

The isolated strain was molecularly identified through analysis of the rDNA locus. Genomic DNA from the strain cultivated in YPD broth was extracted from cell lysate using the AccuPrep^TM^ Genomic DNA Extraction Kit (Bioneer, Daejeon, Korea). The D1/D2 domain of the 26S rDNA gene and the internal transcribed spacer (ITS) region of the rDNA were amplified via polymerase chain reaction (PCR) with Phusion® high-fidelity DNA polymerase (NEB, Ipswich, MA, USA) and appropriate primer pairs, using the isolated strain's genomic DNA as the template. For PCR amplification of rDNA locus genes from eukaryotic strains, the primers used in this study were as follows: NL-1 (5′-GCATATCAATAAGCGGAGGAAAAG-3′) and NL-4 (5′-GGTCCGTGTTTCAAGACGG-3′) for 26S rDNA and ITS-1 (5′-TCCGTAGGTGAACCTGCGG-3′) and ITS-4 (5′-TCCTCCGCTTATTGATATGC-3′) (Kim and Kim, 2012; Kim, 2018a). PCR products were purified using the QIAquick® Gel Extraction Kit (Qiagen, Valencia, CA, USA). Purified DNA fragments were cloned into the pGEM-T Easy vector (Promega, Madison, WI, USA) and sequenced. The nucleotide sequences of the D1/D2 26S rDNA domain and the ITS1-5.8S rDNA-ITS4 fragment were deposited into the GenBank database under accession numbers MK691414 and MK685080, respectively. Sequence data were compared to references in public databases, to confirm the identity of the isolated strains using homologous DNA sequences of other *Candida* strains obtained from the NCBI and GenBank databases.

### Morphological Analysis of the Isolated Strain

To confirm the isolated strain as *C. tropicalis*, the strain was streaked onto CHROMagar Candida (BD Diagnostics, Sparks, MD, USA), an indicator plate that incorporates a chromogenic agent. The positive control *C. tropicalis* CBS94 and negative controls *Candida albicans* MYA-682 and MYA-2876 were also streaked onto the indicator plate for comparison of colony colors.

To observe the morphology of the isolated *C. tropicalis* strain, scanning electron microscopy (SEM) analysis was performed at the Korea Research Institute of Bioscience and Biotechnology (Daejeon, Korea) using a Hitachi S4300N (Tokyo, Japan). The freshly cultured strain was fixed in 0.1 M phosphate buffer (pH 7.2) containing 2.5% (v/v) paraformaldehyde-glutaraldehyde for 2 h, and then post-fixed in the same buffer containing 1% (w/v) osmium tetroxide for 1 h. Fixed samples were dehydrated in ethanol, substituted in isoamyl acetate, and then critical point-dried in CO_2_. The prepared samples were sputtered with gold using a Polaron sputter-coater (SC502) and analyzed via SEM.

### Chemotaxonomic Analysis

The isolated yeast was cultivated in YPD medium for 48 h at 30°C until the early exponential phase. Fatty acids were extracted, methylated, and separated using protocols of the MIDI/Hewlett Packard Microbial Identification System. Chemotaxonomic analysis based on the fatty acid composition of the cells was performed by the Korean Culture Center of Microorganisms (Seoul, Korea).

### Physiological Characterization of the Isolated Yeast Strain

The isolated yeast strain was cultivated in YPD broth at 30°C. The culture was subsequently diluted to an optical density at 600 nm (OD600) of 0.1 and 4 additional 6-fold serial dilutions were performed. The cells were spotted on YPD agar plates. The conditions were: YPD with or without chemicals; 0.5, 1.0, or 3.0 M NaCl; 8, 10, or 16 % (v/v) ethanol; 3, 4, 5, or 10 mM H_2_O_2_; 0.25 mM or 0.5 mM calcofluor white; 15, 20, or 25 mM caffeine; and 100 mM xylose. Plates were incubated at 30°C for 3 days. To determine temperature resistance, spotted YPD plates were incubated at 25, 30, 37, 42, and 45°C.

For the carbohydrate utilization test, the yeast strain was freshly cultivated in YPD for 24 h, and then 1/1,000 of the culture volume was inoculated in minimal medium containing 2% (w/v) of the sole carbon source and 0.67 % (w/v) yeast nitrogen base without amino acids. The sole carbon sources used in this study include hexoses: D-fructose, D-glucose, D-galactose, D-mannose, and D-rhamnose; pentoses: L-arabinose, D-arabinose, D-xylose, and D-ribose; disaccharides: lactose, cellobiose, sucrose, maltose, melibiose, and trehalose; the trisaccharide D-raffinose; polysaccharides: starch, carboxymethylcellulose, avicel, xylan oat spelt, xylan birch wood, xylan beech wood, and inulin; sugar alcohols: glycerol, D-sorbitol, D-mannitol, and D-xylitol; sugar acids: D-gluconic acid, D-xylonic acid, D-glucuronic acid, and D-galactonic acid; alcohols: methanol and ethanol; and the amino sugar N-acetylglucosamine. Cells inoculated in each culture broth were cultivated at 30°C with 240 rpm shaking.

### Shaking Flask Cultivation for Xylitol Production

For the xylitol production test supplemented with co-substrate, the yeast strain was cultivated in YPD, and then the culture was inoculated in basal medium (2% (w/v) xylose and 0.67% (w/v) yeast nitrogen base) supplemented with 2% (w/v) of an additional carbon source, D-glucose; D-galactose; D-mannose; D-fructose; D-xylose; D-sucrose; D-maltose; inulin; D-sorbitol; D-mannitol; D-trehalose; or D-raffinose. The inoculated culture broths were cultivated at 30°C with 240 rpm shaking.

To test resistance against high concentrations of xylose for xylitol production, the yeast cell culture was inoculated in semi-defined medium composed of various concentrations of xylose (100, 200, 300, 400, 500, and 600 g/L), with 0.5% (w/v) yeast extract, 0.5% (w/v) KH_2_PO_4_, 0.5% (NH_4_)_2_SO_4_, and 0.04% MgSO_4_·7H_2_O, adjusted to pH 6.0. The cells were cultivated at 30°C with shaking at 150 rpm.

Cell growth and xylitol concentrations were monitored at 24-h intervals. The cell growth (OD600) was measured with a spectrophotometer based on the absorbance at 600 nm. The culture broth was centrifuged and the supernatant was analyzed for xylitol content through high-performance liquid chromatography (HPLC), as described below.

### Batch Bioreactor Cultivation for Xylitol Production

For xylitol production, two xylose sources were used. One source was pure xylose chemical reagent, and the other was xylose solution prepared from the first dilute acid pretreatment of EPFBFs in the sequential acid-/alkali-pretreatment process (Kim et al., 2012a; Kim, 2019). The isolated *C. tropicalis* was cultivated in semi-defined medium containing 2% (w/v) xylose for 24 h, as described above. A 10% (v/v) seed culture of the yeast was inoculated into a 1-L fermenter FMT ST-S (Fermentec, Cheongju, Korea), with a 0.5-L working volume and basal medium containing 100 g/L (w/v) pure xylose or a solution of approximately 74 g/L xylose prepared from the biomass acid hydrolysate supplementing the basal medium. The fermenter was operated at 30°C with stirring at 150 rpm.

### Cloning and Sequence Analysis of the Xyl1, Xyl2, and Xyl3 Genes

To clone the gene family responsible for initial xylitol metabolism in *C. tropicalis*, the xylose reductase (XR)-encoding *xyl*1, xylitol dehydrogenase-encoding *xyl*2, and xylulose kinase-encoding *xyl*3 genes were directly amplified from genomic DNA prepared using the protocol described above. Each gene was amplified through PCR with Phusion® high-fidelity DNA polymerase and the appropriate primer pairs using genomic DNA as the template. The primers used for gene amplification were as follows: xyl1-F (5′- ATGTCTACTACTCCTACTATTCCTAC-3′) and xyl1-R (5′-TTAAACAAAGATTGGAATGTTGTCCC-3′) for the *xyl*1 gene, xyl2-F (5′-ATGACTGCAAACCCATCATTAGTTC-3′) and xyl2-R (5′- CTATTCTGGACCATCAATTAAAC-3′) for the *xyl*2 gene, and xyl3-F (5′- ATGACTACTGATTATTCTGAAAACGAC-3′) and xyl3-R (5′- TTATTGTTTTAATAAAGTCTCTTCC−3′) for the *xyl*3 gene. The cloning procedures for the amplified genes followed the protocol described above for cloning of D1/D2 26S rDNA and ITS1-5.8S rDNA-ITS4 genes. The nucleotide sequences of the *xyl*1, the *xyl*2, and the *xyl*3 genes were deposited into the GenBank database under accession numbers MK690391, MK690392, and MK690393, respectively.

### Computational Homology Modeling of Xylose Reductase With Xylose and NADH

As no xylose reductase (XR) molecular structures from *C. tropicalis* strains have yet been revealed, structure homology modeling was performed using the pseudo-quadratic restraints with simulated annealing (PQR-SA) method (Kim et al., 2012b). The structural qualities of two structures obtained through homology modeling were validated using various quality parameters evaluated with PROCHECK (Laskowski et al., 1993), WHATCHECK (Vriend, 1990), and MOLPROBITY (Chen et al., 2010). As a protein structure template, *Candida tenuis* xylose reductase (CtXR) (Protein Data Bank entry code: 1MI3) was used for the homologous protein models (Kratzer et al., 2006). Ligand-bound protein structures were also constructed using CtXR as a template, with nicotinamide adenine dinucleotide phosphate (NADPH) as a cofactor (PDB entry code: 1K8C). The structures of CtXR and the *C. tropicalis* counterpart proteins were superimposed to compare their structural differences, using both CHARMM (Chemistry at HARvard Marcomolecular Mechanics) (Brooks et al., 2009) and the TM-align program (Zhang and Skolnick, 2005). The comparison of the superimposed NADPH-bound XR structures was evaluated using Jmol (http://www.jmol.org/).

### Cloning, Site-Directed Mutagenesis, Enzyme Production, and Purification

To produce recombinant XR, the XR-encoding *xyl*1 gene fragment was amplified via PCR using the cloned vector described above and the appropriate primer set. The PCR products were digested with restriction enzymes, and the resulting DNA fragments were cloned into the expression vector pET39b to yield pET-XR. The plasmid contained an N-terminal 10xHis coding gene tag for purification. Finally, the recombinant vector was transformed into the *E. coli* strain BL21(DE3)pLysS.

To introduce the site-directed mutation into the *xyl*1-encoding gene, the plasmid pET-XR was used as a template. The oligonucleotide primers used for PCR in the forward direction were designed to generate S279L or S279N mutants. The primers for the reverse direction also contained mismatched bases for mutations (relevant bases are underlined): S279N, 5′-GAAACATTGCTGTTATTCCAAAATCAAACAATCCAGAAAGATTAGCTC-3′ (forward) and 5′- GAGCTAATCTTTCTGGATTGTTTGATTTTGGAATAACAGCAATGTTTC-3′ (reverse); S279L, 5′-GAAACATTGCTGTTATTCCAAAATCAAACTTCCAGAAAGATTAGCTCAAAAC-3′ (forward) and 5′-GTTTTGAGCTAATCTTTCTGGAEGTTTGATTTTGGAATAACAGCAATGTTTC-3′ (reverse). The primer melting temperature (T_*m*_) and the optimum length of the primers were calculated using the Quick Change® primer design program from Stratagene (Stratagene, La Jolla, CA, USA). The 50-μL PCR reaction was carried out with 50–100 ng template, 0.2–2 μM primers, 200 μM dNTPs, and 2.5 U of *Pfu Turbo* DNA polymerase (Stratagene). The products of PCR amplification were treated with the restriction enzyme DpnI (Fermentas) for 2 h at 37°C. An aliquot of 5 μL of the PCR product described above was transformed into *E. coli* DH5α chemically competent cells and inoculated on a Luria-Bertani (LB) plate containing 30 mg ml^−1^ kanamycin. Several colonies were selected and their plasmids were isolated using plasmid mini-prep. All mutants were confirmed through DNA sequencing.

To produce recombinant XR and its mutant enzymes, recombinant *E. coli* strains harboring the recombinant plasmids were cultivated in Terrific Broth (TB) medium supplemented with 30 μg/mL kanamycin at 28°C for 24 h, and 0.5 mM IPTG was then added to the culture broth to induce lectin gene expression. After further cultivation at 25°C for 8 h, the cells were harvested, resuspended in lysis buffer [10 mM Tris-HCl (pH 8.0) containing 10 mM imidazole, 250 mM NaCl, 1 mM PMSF and EDTA-free protease inhibitor cocktail (Sigma-Aldrich, St. Louis, MO, USA)], and then disrupted with three passes through a French press at > 1,000 psi. The crude lysate was centrifuged at 100,000 × g for 1 h at 4°C. Recombinant proteins from the clarified homogenate were purified through a three-step chromatography procedure using affinity chromatography HisTrap FF, ion-exchange chromatography HiTrap Q FF, and gel-filtration chromatography Superose 12 columns. Finally, the purified recombinant protein was analyzed using tricine-PAGE, pooled, and concentrated through ultrafiltration.

### Enzyme Assay of Recombinant Xylose Reductase

Activities of the recombinant XR and its mutant enzymes were determined spectrophotometrically through monitoring changes in the absorbance at 340 nm, representing the oxidation of NAD(P)H. The enzymatic reaction was carried out at 30°C in 50 mM TrisHCl buffer (pH 6.8) containing 10 mM xylose, 10 mM NAD(P)H, 1 mM β-mercaptoethanol, and an appropriate concentration of the purified enzyme. Each activity determination was performed in triplicate in enzyme reaction mixtures obtained at different times. One unit of XR activity is defined as the amount of enzyme required to catalyze the formation of 1 μmol NADP^+^ per min using an extinction coefficient of 6.22 ×10^−3^ M^−1^·cm^−1^.

### Analytical Methods

Levels of xylose, other sugars, and organic acids were determined using an HPLC system equipped with a refractive index detector, an auto-sampler, and an Aminex HPX-87P column (Bio-Rad, Hercules, CA, USA) for monosaccharide analysis, or a Rezex ROA-Organic Acid H+ column (Phenomenex Inc., Torrance, CA, USA) for organic acid analysis, as described previously (Kim, 2018b,c, 2019). All samples were clarified through filtration with a 0.20-μm filter and then injected into the analytical HPLC column. All analyses were performed in triplicate.

## Results

### Isolation of a Strain With Stress Tolerance to Acid Pretreatment Wastewater

Honey, with its high sugar content, was a target resource for isolation of a stress-tolerant strain. Honey contains mainly carbohydrates, such as fructose, glucose, and other sugars, as well as organic acids including acetic, butanoic, formic, citric, succinic, lactic, malic, pyroglutamic and gluconic acids, and a number of aromatic acids. Additionally, honey contains hydroxymethylfurfural (HMF), a common product of hexose degradation below pH 5 (Zirbes et al., 2013). Honey was sampled with a loop and inoculated through streaking onto minimal medium supplemented with neutralized EPFBF acidic hydrolysate solution (Kim, 2019) as the sole carbon source. The culture broth was subsequently cultivated five times and a strain from the final culture was selected that showed similar colony morphology on a solid agar plate. Colonies on the plate appeared as thick, milky white yeast (data not shown).

### Phylogenetic and Taxonomic Identification

To identify the isolated strain, the 26S rDNA D1/D2 domain and ITS1-5.8S rDNA-ITS4 region were amplified from the microorganism's genomic DNA, forming individual bands of 1,112 bp for the conserved 26S rDNA D1/D2 domain and 538 bp for the ITS1-5.8S rDNA-ITS4 locus. After DNA sequencing, comparison of the PCR-amplified products to reference sequences in GenBank revealed the 5.8S rDNA of *Candida* species. The isolated strain showed about 99% identity with *C. tropicalis* strains AN1 (EU924133.1), CBL Cd-3 (EU924133.1), LMICRO522 (KF746430), greater than that with other *Candida* species. Analysis of the ITS1-5.8S rDNA-ITS4 locus sequence and a phylogenetic tree constructed on the basis of these sequences revealed highly conserved relationships and tight clustering among *C. tropicalis* strains (Figure S1A). On the other hand, when 26S rDNA D1/D2 domain sequences were compared, the isolated yeast strain displayed 100% identity with *C. tropicalis* strain ATCC750 (AB457174). This sequence also showed 99% identity with those of other *Candida* species, including *C. tropicalis* Ph43 (JN940623), *C. albicans* (GQ495089), *C. parapsilosis* (JN940627), *C. tropicalis* CBS94 (AY497695), *C. maltosa* (AY653546), and *Lodderomyces elongisporus* (AY653547) (Figure S1B). In contrast to 26S rDNA D1/D2 domain sequence analysis, the phylogenetic tree based on the ITS1-5.8S rDNA-ITS locus of the isolated yeast strain clearly showed close relationships with the other three *C. tropicalis* strains (Figure S1A). Analysis of the conserved ITS1-5.8S rDNA-ITS4 locus and 26S rDNA D1/D2 domain confirmed that the isolated yeast strain was *C. tropicalis*, and this strain was named YHJ1.

In chemotaxonomic characterization, the major fatty acids of the isolated yeast strain were C_18:1_ cis9 (ω9) (54.28%), C_18:2_ cis9 (9.79%), C_16:0_ (15.33%), and C_16:1_ cis9 (ω7) (11.57%). The minor fatty acids were C_18:0_ (5.37%), C_17:0_ (0.62%), C_17:1_ (1.57%), C_15:0_ (0.35%), C_14:0_ (0.86%), and C_12:0_ (0.27). The fatty acid composition was significantly different from those of other *Candida* stains (Peltroche-Llacsahuanga et al., 2000). The phylogenetic and chemotaxonomic results supported the isolated yeast as a *C. tropicalis* strain distinct from *C. albicans* or other known *Candida* species.

### Morphological Analysis of the Isolated Yeast Strain

In SEM analysis of morphology, the isolated strain appeared as blastoconidia in a budding-yeast phase of growth (Figure S2A). The budding yeast revealed a smooth and homogenous cell surface with bud scars located randomly. No tuber-like, pseudohyphal, or filamentous forms were detected in the isolated yeast cultivated in rich medium.

To confirm the comparison of the isolated yeast strain with other *Candida* species, the strain was streaked on a BBL CHROMagar Candida plate, which can be used to identify and recognize *Candida* species, including *C. tropicalis, C. albicans*, and *C. krusei*, through the formation of different colony colors on the indicator plate (Figure S2B). The isolated yeast strain showed deep metallic blue colonies compared to the positive control *C. tropicalis* CBS94. On the other hand, *C. albicans* MYA-682 and MYA-2876 showed pale blue and deep green colors on the plate, respectively. This indicating plate clearly revealed that the isolated yeast strain belongs to the species *C. tropicalis* rather than *Candida albicans*.

### Characterization of Strain Tolerance Toward Different Chemical Reagents

**Figure 1:**
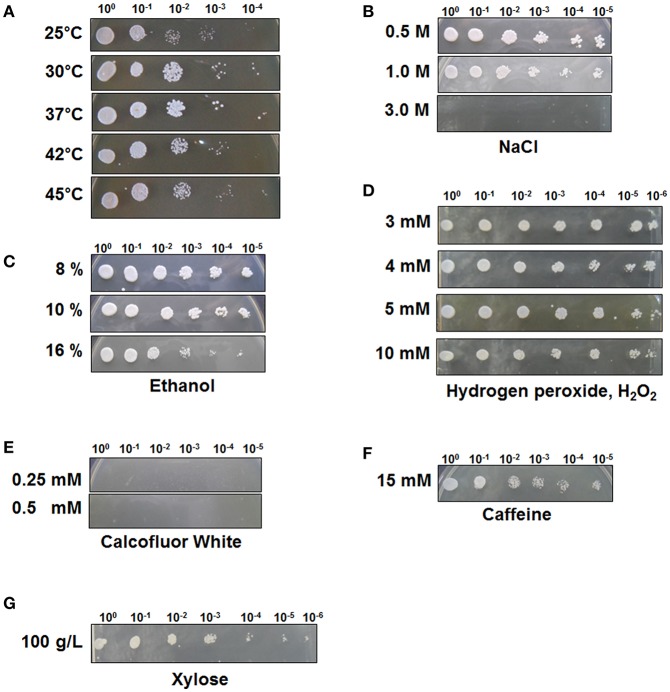
*C. tropicalis* YHJ1 shows different resistance and sensitivity patterns to different chemicals in the spot dilution assay. The culture was diluted to an optical density at 600 nm (OD600) of 0.1 and then four additional 6-fold serial dilutions were made. The cells were spotted on YPD agar plates. **(A)** Temperature resistance was tested by incubating the spotted YPD plates at 25, 30, 37, 42, and 45°C. The conditions were: YPD with or without chemicals: **(B)** 0.5, 1.0, and 3.0 M NaCl; **(C)** 8, 10, and 16 % (v/v) ethanol; **(D)** 3, 4, 5, and 10 mM H_2_O_2_; **(E)** 0.25 mM and 0.5 mM calcofluor white; **(F)** 15 mM Caffeine and **(G)** 100 mM xylose. Plates were incubated at 30°C for 3 days.

To test the stress tolerance of *C. tropicalis* YHJ1, spot-dilution assays were performed with different chemical reagents at 30°C, as the pre-spotting assay for the yeast revealed optimal growth at 30°C among the tested temperatures of 25, 30, 37, 42, and 45°C (Figure 1A). The spot-dilution assay results showed the relative tolerance of the strain to each chemical agent at different concentrations (Figures 1B–G). The chemical stress responses of the isolated yeast showed differences in sensitivity and resistance against different reagents. *C. tropicalis* YHJ1 revealed chemical stress resistance against NaCl, ethanol, hydrogen peroxide, caffeine, and high concentrations of xylose, but not calcofluor white. The isolated yeast could grow on agar medium containing up to 1.0 M NaCl, 16% (w/v) ethanol, 10 mM H_2_O_2_, and 15 mM caffeine. In addition, the strain showed a normal growth pattern in agar medium containing a high xylose content (100 g/L). Therefore, its growth was further evaluated under liquid culture conditions with a high xylose concentration. On the other hand, the strain was sensitive to high concentrations of calcofluor white, which might lead to inhibition of chitin and β(1, 3)-glucan synthesis for yeast cell wall formation (Roncero and Durán, 1985).

### Effects of Xylose Concentration and Co-substrate Addition on Xylitol Production

**Figure 2:**
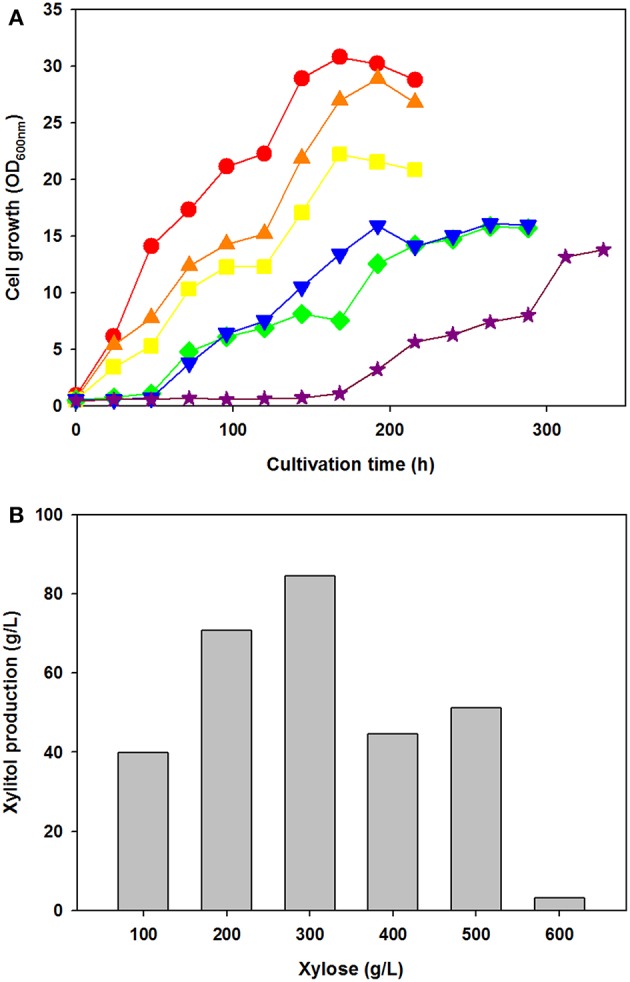
Effect of xylose concentration on yeast cell growth **(A)** and maximum xylitol production **(B)** in semi-defined medium. **(A)** Cell growth was monitored through measurement of the absorbance at 600 nm using a spectrophotometer. The initial xylose concentration in the culture medium was 100 (circle), 200 (triangle), 300 (square), 400 (reverse triangle), 500 (diamond), or 600 (star) g/L. **(B)** The maximum xylitol concentration was analyzed using high-performance liquid chromatography (HPLC). All analyses were performed in triplicate. The data are expressed as mean ± standard error of the mean, *n* = 3.

To test the effect of xylose concentration on cell growth and xylitol production, *C. tropicalis* YHJ1 was cultivated in defined medium containing various concentrations of xylose, ranging from 100 to 600 g/L under aerobic conditions (Figure 2). Interestingly, the initial cell growth rates were faster, with lower concentrations of xylose (Figure 2A). No lag phase of growth was detected in cell cultures with 100–300 g/L xylose, whereas the cells cultivated with 400–500 g/L xylose showed a 48-h lag phase. Moreover, cells cultivated with 600 g/L xylose revealed a 144-h lag phase. Maximum xylitol production showed different patterns from the cell growth rates in medium with various xylose concentrations (Figure 2B). The highest xylitol concentration was detected in the yeast culture with 300 g/L xylose, whereas the lowest level was observed in the culture medium with 600 g/L xylose. In addition, maximum xylitol production increased gradually among the yeast cultures with 100–300 g/L xylose, whereas sugar alcohol production decreased dramatically in medium supplemented with over 400 g/L sugar. A high concentration of xylose might cause sugar metabolism to convert xylose into xylitol in the cells.

**Figure 3:**
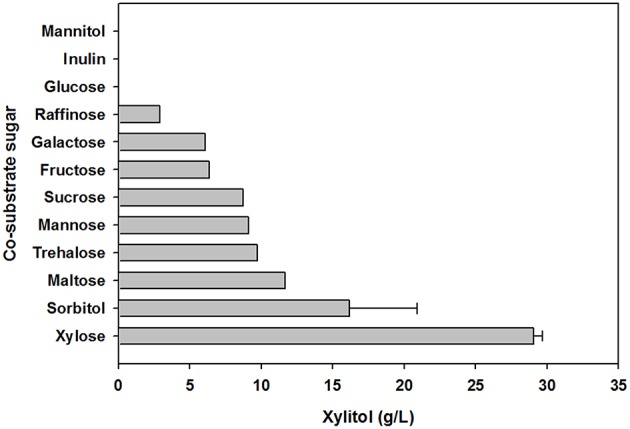
Effect of carbohydrate co-substrates on xylitol production. The xylitol concentration was analyzed using HPLC. All analyses were performed in triplicate. The data are expressed as mean ± standard error of the mean, *n* = 3.

To evaluate the effect of co-substrate addition on xylitol production, an equal amount of another sugar was added to the yeast cell culture medium, in addition to xylose. In preliminary tests of carbohydrate utilization, *C. tropicalis* YHJ1 could metabolize hexose sugars (fructose, glucose, galactose, and mannose), pentose sugars (xylose), disaccharides (cellobiose, sucrose, maltose, and trehalose), trisaccharide (raffinose), sugar acids (gluconate and xylonate), sugar alcohols (glycerol, sorbitol, mannitol, and xylitol), and an amino sugar (N-acetylglucosamine) in minimal medium for cell growth (Table S1). Among the utilizable carbohydrate substrates, 11 candidates were selected and individually added to defined medium containing 2% (w/v) xylose. After 96 h cultivation, the highest xylitol production was detected in cell culture supplemented with only xylose and no other sugar (Figure 3). No xylitol was detected in yeast cultures containing glucose or inulin. Nevertheless, the maximum cell growth was observed in medium containing sucrose (data not shown). These data indicate that xylitol production did not depend on cell growth, and that xylose metabolism could be tightly regulated by certain carbohydrates.

### Xylitol Fermentation Using Xylose Solution Prepared From Acid Pretreatment Wastewater

**Figure 4:**
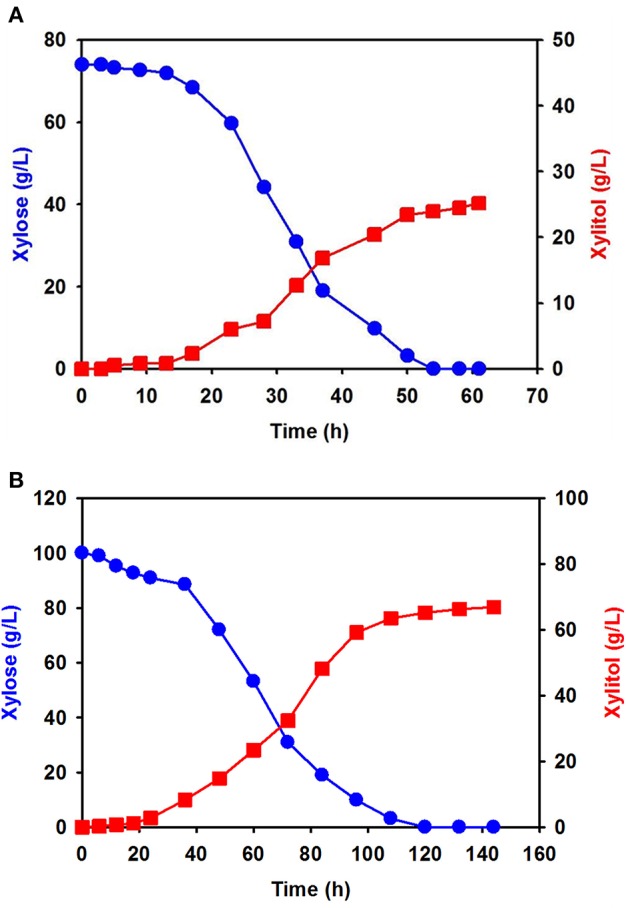
Batch fermentation for xylitol production by *C. tropicalis* YHJ1 in a 1-L jar fermenter. Xylitol fermentation was performed using xylose solution prepared from acidic pretreatment wastewater **(A)** and pure xylose **(B)** in the semi-defined medium described in the materials and methods section. The amounts of xylose (circles) and xylitol (squares) were analyzed using HPLC. All analyses were performed in triplicate.

Using xylose solution obtained from acidic pretreatment wastewater, batch fermentation was performed for xylitol production with the isolated *C. tropicalis* YHJ1 in a 1-L jar fermenter. The initial concentration of xylose in the semi-defined medium was 74.0 ± 0.8 g/L. After inoculation of the yeast, xylitol fermentation proceeded gradually for 60 h (Figure 4A). The concentration of xylose decreased gradually due to cell growth. All xylose in the culture broth was depleted at 54 h. The highest xylitol concentration was 25.2 ± 0.5 g/L at 60 h, giving a xylitol yield of 0.41 g xylitol/g xylose at 60 h.

Batch fermentation in medium supplemented with 100 g/L of pure xylose was also carried out under the same culture conditions. Interestingly, xylose consumption and xylitol production were slower than in the previous batch culture (Figure 4B): xylitol production was slow until 22 h, indicating a lag phase, and then increased exponentially, entering a stationary phase between 100 and 140 h. The xylose consumption pattern was the opposite of that for xylitol production in the culture. Although batch fermentation required almost double the time, the maximum xylitol production was 66.9 ± 1.2 g/L at 142 h, giving a xylitol yield of 0.67 g xylitol/g xylose at 142 h.

### Cloning and Characterization of Genes Involved in Initial Xylose Metabolism

To identify the genes involved in initial xylose metabolism in *C. tropicalis* YHJ1, the *xyl*1, *xyl*2, and *xyl*3 genes were directly amplified from its chromosomal DNA through PCR, using oligonucleotides from the NCBI database for these genes in other *C. tropicalis* strains. The amplified DNA fragments were cloned and sequenced. The deduced amino acid sequences of *C. tropicalis* YHJ1 XYL1, XYL2, and XYL3 showed 99% identity with the NADPH-dependent D- XR II/III protein (NCBI accession numbers, XP_002546500.1) of *C. tropicalis* MYA-3404, 99% identity with the xylitol dehydrogenase (ABG49459.1) of *C. tropicalis* Ct1799, 99% identity with a hypothetical protein CTRG_03873 (XP_002549576.1) of *C. tropicalis* MYA-3404, and 80% identity with the D-xylulokinase (AAY87404.1) of *C. maltose* Xu316. The nucleotide sequences of the *C. tropicalis* YHJ1 *xyl*1, *xyl*2, and *xyl*3 genes were deposited in GenBank under accession numbers MK690391, MK690392, and MK690393, respectively.

### Computational Structure Homology Modeling for Substrates Bound by Xylose Reductase

**Figure 5:**
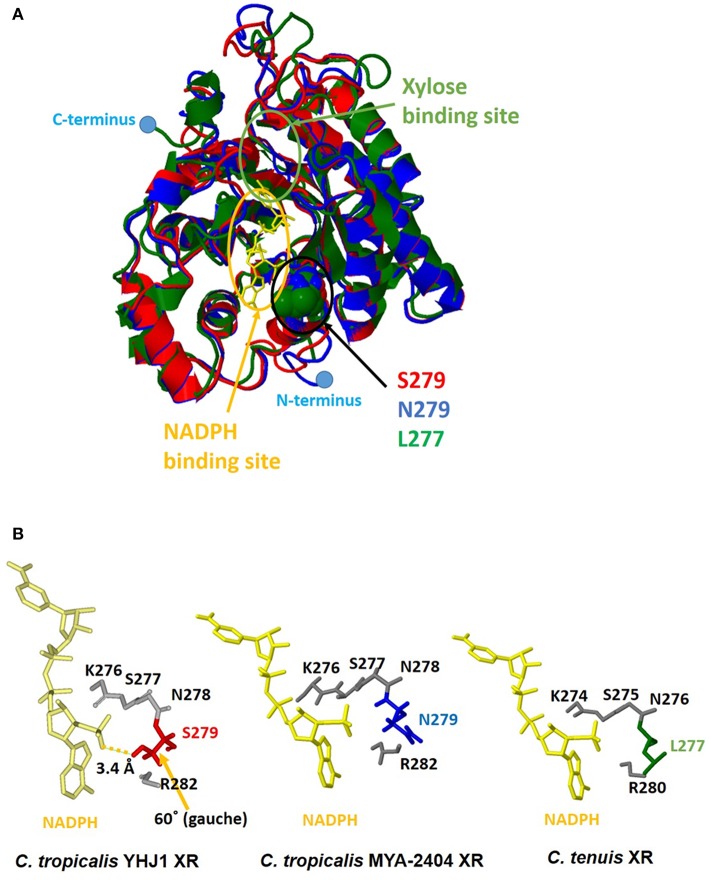
Computational homologous protein modeling for xylose reductase (XR). **(A)** Three superimposed structures drawn as a three-color ribbon diagram (red, *C. tropicalis* YHJ1 XR; blue, *C. tropicalis* MYA-2404 XR; green, *C. tenuis* XR). The xylose and nicotinamide adenine dinucleotide phosphate (NADPH) binding sites are indicated by green and yellow circles, respectively. The cofactor NADPH is represented by a yellow stick model. The active site domain is indicated by a black circle and three different amino acids are shown as space-filling models. The C- and N-termini are marked with blue dots. **(B)** The studied active sites of the three structures from *Candida* XR proteins. The NADPH cofactors and active sites are shown as yellow and gray stick models. Three different amino acids are shaded in red for *C. tropicalis* YHJ1 XR, blue for *C. tropicalis* MYA-2404 XR, and green for *C. tenuis* XR.

Although the amino acid sequence (XYL1) of *C. tropicalis* YHJ1 XR has 99% identity with counterpart proteins in other *Candida* strains, three variable residues in its sequence, Asn279Ser, Lys296Gln, and Asp297Glu, were observed through comparison with the *C. tropicalis* MYA-2404 XR sequence. To determine whether these variable residues could interact with or bind to xylose or a coenzyme acting as a co-substrate NADPH, structure homology modeling was performed. The protein crystal structure (PDB accession number 1MI3) of CtXR (green) (Kratzer et al., 2006) was used to construct the homologous protein model for *C. tropicalis* YHJ1 XR (red) and *C. tropicalis* MYA-2404 XR (blue). Homology modeling with *C. tenuis* XR (Protein Data Bank entry code: 1MI3) revealed sequence identity of 0.78 for *C. tropicalis* YHJ1 XR and 0.76 for *C. tropicalis* MYA-2404 XR. Three superimposed structures of *Candida* XR proteins were constructed (Figure 5A). These three structures are well-conserved, with the same secondary structures, but their coil regions differ slightly. The active sites that can interact with the phosphate group of NADPH are located near NADPH. Of the three variable amino acid residues listed above in the active site of the homologous structures, the position corresponding to the Ser279 residue in *C. tropicalis* YHJ1 XR was observed as Asn279 in *C. tropicalis* MYA-2404 and Leu277 in *C. tenuis* XR proteins. The Ser279 residue may interact tightly with the phosphate moiety in NADPH within 3.4 Å, blocking the coenzyme from stabilizing the transient enzyme-substrate complex, in contrast to its two counterpart proteins (Figure 5B).

On the other hand, the Asn279 residue in *C. tropicalis* MYA-2404 XR may interact repulsively with the positively charged phosphate group, which might destabilize the binding of NADPH to the protein. In addition, the Leu277 residue in *C. tenuis* XR has a non-polar tendency, causing weaker binding of NADPH compared to the other residues in *C. tropicalis* XR structures. Thus, the Asn279 or Leu277 residue might fail to bind or interact with the non-phosphorylated cofactor NADH due to the long distance between the ligand and the side chain of the amino acid.

### Comparison of the Catalytic Activities of Xylose Reductase and Its Mutants

To confirm that the Ser279 residue in *C. tropicalis* YHJ1 XR affects catalytic activity, two mutants of the wild-type XR containing Ser279Asn or Ser279Leu were constructed. The his-tag fused recombinant wild-type *C. tropicalis* XR (XR_S279) and these two mutants (XR_S279L and XR_S279N) were produced under identical culture conditions, as described in the materials and methods section. Analysis through tricine-PAGE, as well as western blotting of the induced *E. coli* extract, showed dense bands of expressed protein at the expected position of approximately 34 kDa (Figure S3). The wild-type and mutant proteins were purified using the affinity chromatography and sequential column chromatography techniques described in the materials and methods section.

The specific enzyme activities of the wild-type and mutant XR enzymes were determined using NADPH or NADH as a cofactor, and these data are summarized in Table 1. The specific activities of wild-type XR were 10.1 ± 1.2 U/g protein for NADPH and 1.3 ± 0.2 U/g protein for NADH. Thus, the enzyme activity of the wild-type XR toward NADPH was 7.8-fold higher than that toward NADH. The XR_S279L and XR_S279N mutant enzyme activity was 7.3 ± 0.5 U/g protein and 6.9 ± 0.3 U/g protein toward NADPH, respectively. These mutant enzymes also exhibited higher activity toward NADPH than NADH. Comparison of specific activities revealed that the wild-type XR of the stress-tolerant *C. tropicalis* YHJ1 strain has higher activity toward the phosphorylated cofactor NADPH, by approximately 1.4-fold, compared to XR_S279L and 1.5-fold for XR_S279N.

**Table 1:** Enzyme activities of *C. tropicalis* YHJ1 xylose reductase and its mutant proteins.

**Cofactor/enzyme [specific activity]**	**Wild-type XR**	**XR_S279L**	**XR_S279N**
	**[U/g protein]**	**[U/g protein]**	**[U/g protein]**
NADPH	10.1 ± 1.2	7.3 ± 0.5	6.9 ± 0.3
NADH	1.3 ± 0.2	1.0 ± 0.1	1.2 ± 0.2

## Discussion

In biorefinery processes using a renewable bioresource, biomass pretreatment is a key step for turning degradable biopolymers into fermentable sugars. Depending on the biomass materials employed, numerous approaches using physical, chemical, biological, or hybrid methods have been applied to optimize the treatment process by lowering cost and energy consumption (Melin and Hurme, 2011; Brethauer and Studer, 2015; Caicedo et al., 2016). Among these pretreatment technologies, chemical pretreatments using acid or alkali reagents have been used due to their relatively low cost and easy operation (Melin and Hurme, 2011; Kim et al., 2013; Kim and Kim, 2014; Capolupo and Faraco, 2016; Liu et al., 2017). Despite these benefits, such pretreatment processes generate wastewater containing lignin and hemicellulose fractions solubilized from lignocellulosic materials, which demand further treatment (Abdolali et al., 2014; Kim, 2019). The wastewater from chemical pretreatment usually contains monomerized sugars and thermally degraded products, e.g., furfural, acetic acetate, and aliphatic and aromatic compounds. These chemical compounds in biorefinery products may be extracted or purified from the wastewater for further application. Indeed, the separated chemical wastewater still contains acidic or basic chemical catalysts, and thus could be recycled and reused for pretreatment of further biomass. In our previous research, sequential acid-/alkali-pretreatment was used to increase the cellulose content in EPFBFs, an agricultural byproduct (Kim et al., 2012a; Kim and Kim, 2013); recycling and reuse of the acidic pretreatment wastewater allowed accumulation of the hemicellulose fraction from EPFBF in the biomass hydrolysate solution. Furthermore, acid-hydrolyzed xylose prepared from post- pretreatment acidic wastewater could be fermented to produce xylitol using an adapted yeast strain (Kim, 2019).

In this study, we used neutralized acid EPFBF hydrolysate to isolate a stress-tolerant strain for application in xylose fermentation to produce sugar alcohol. Honey was used as a candidate resource for isolation of a stress-tolerant strain that could utilize xylose, a fermentable sugar found in the acidic wastewater from sequential acid-/alkali-pretreatment of biomass. Although honey is primarily made up of sugars, sugar acids, and HMF are also present as minor compounds (Zirbes et al., 2013; Islam et al., 2014). Among these minor compounds, HMF is a fermentation-inhibitory compound generated from hydrolyzed sugars under high-temperature, acidic conditions (Jönsson and Martín, 2016; Wang et al., 2018). The strain isolated from honey was identified as a xylose-metabolizing yeast, *C. tropicalis* YHJ1, through phylogenetic, taxonomic, and morphological analysis (Figures S1, S2 and Table S1). This yeast displayed stress tolerance to various chemical reagents, including NaCl, ethanol, hydrogen peroxide, caffeine, and high concentrations of xylose, but not to calcofluor white, an inhibitor of yeast cell wall construction (Figure 1). Interestingly, *C. tropicalis* YHJ1 showed a strongly a resistant phenotype to high concentrations of xylose up to 600 g/L, despite reduced cell growth dependent on the sugar content in the culture medium (Figure 2A). The resistance capacity of the yeast was not correlated with xylitol production from xylose metabolism (Figure 2B). The critical concentration for cell growth and xylitol production was 300 g/L xylose. Morphological changes were observed in the budding yeast at concentrations over 300 g/L xylose (Figure S4). Interestingly, rough, heterogeneous, and tuber-like cell populations increased in cultures with higher xylose concentrations. These morphological changes might be related to xylose or other cellular metabolic pathways. These phenotypic switching in *C. tropicalis* has been known as a virulence event for colonization and infection of different host niches (Homann et al., 2009; Porman et al., 2011). In addition to these morphological changes in the isolated *C. tropicalis* strain under high concentration of xylose, the spotting assays showed calcofluor white, a chitin synthase inhibition, inhibited strongly the cell growth (Figure 1E). These results imply that the isolated yeast might be a potential pathogenic Candidate strain.

In the carbohydrate utilization test using minimal medium supplemented with a single carbon source, the isolated strain showed similar utilization patterns for various hexose and pentose sugars, disaccharides, polysaccharides, and sugar alcohols to those of the counterpart stain *C. tropicalis* CB92 (Table S1). However, *C. tropicalis* YHJ1 showed greater utilization capacity for xylitol than strain CB92. The isolated yeast might have more effective enzymes or transporters for xylitol metabolism. In addition to carbohydrate utilization, *C. tropicalis* YHJ1 produced the highest xylitol concentration in medium supplemented with only xylose, and no other carbohydrate co-substrates (Figure 2). Cells cultivated in medium supplemented with glucose or a glucose moiety containing a polysaccharide, inulin, did not produce any xylitol. Interestingly, mannitol and its oxidized form mannose, showed differing metabolism patterns, with cells cultivated in medium containing mannitol rather than mannose appearing to strongly control genes for carbohydrate metabolism through catabolic repression. These data suggest that the initial step of xylose metabolism for xylitol production, involving the conversion of xylose to xylulose, could be tightly regulated in yeast (Goli et al., 2012).

Batch fermentation using a xylose solution prepared with acid pretreatment wastewater from sequential acid-/alkali-pretreatment of EPFBFs showed a lower xylitol concentration and 40% lower yield compared to the culture supplemented with pure xylose (Figure 4). The xylose solution obtained from biomass pretreatment wastewater contained thermally degraded substances, such as acetic acid, furfural, organic acids, and aliphatic and aromatic compounds derived from the lignin and hemicellulose fraction of EPFBFs. Furfural, a minor compound in the sugar solution prepared from acidic pretreatment wastewater, could directly affect oxidoreductases that mediate redox potential in the xylitol metabolism pathway in cells (Modig et al., 2002). In biorefinery production applications using several different biomass hydrolysates, these thermally degraded compounds have been reported to negatively influence cell growth and metabolism (Liu, 2011; Thompson et al., 2016; Wang et al., 2018). The organic acids and phenolic compounds present are also known inhibitors of XR and xylitol dehydrogenase, the initial enzymes involved in xylose metabolism in several *Candida* strains (Lima et al., 2004; Zhang et al., 2012a,b; Wang et al., 2013).

During the initial step of xylose metabolism in *Candida* strains, a transported xylose is first reduced by NADPH- or NADH-dependent XR, encoded by the gene *xyl*1, to create xylitol. Xylitol, a sugar alcohol, is then oxidized by NADP^+^- or NAD^+^-dependent xylitol dehydrogenase, encoded by the gene *xyl*2, to form xylulose. Xylulose is further metabolized to xylulose-5-phosphate by ATP-dependent xylose kinase, encoded by the gene *xyl*3. Subsequently, this phosphate sugar enters the pentose-phosphate pathway. The deduced amino acid sequences of the *xyl*1, *xyl*2, and *xyl*3 genes involved in xylose metabolism in *C. tropicalis* YHJ1 had 99% identity to their counterpart sequences reported from other *C. tropicalis* strains. Nevertheless, XR, the enzyme mediating the first step of xylitol production, has a different amino acid residue in *C. tropicalis* YHJ1, which could influence the tight interaction between the cofactor NADPH and the active site of the protein, as shown through structure homology modeling (Figure 5). In addition to superimposed structure modeling with the ligand, the catalytic activity of the recombinant wild-type XR_S279 toward NADPH was clearly higher than those of the other two mutants, XR_S279L and XR_S279N. Based on XR structure modeling, the Ser279 residue in *C. tropicalis* YHJ1 XR might closely interact with the phosphate moiety of NADPH, but this would not occur with Leu279 or Asn279 in the putative cofactor binding pocket. Although the overall amino acid sequences of XRs do not significantly differ among *Candida* strains, minor sequence differences near enzyme active sites might affect the catalytic activities as well as the selectivity for a phosphate or non-phosphate cofactor. However, further enzymatic kinetics studies are needed to evaluate the catalytic activities of the wild-type XR and other mutants with other non-phosphorylated cofactors and compounds that are inhibitory to xylitol production.

## Conclusion

The xylose-fermenting yeast *C. tropicalis* YHJ1 was isolated from honey and molecularly identified based on phylogenetic, taxonomic, and morphological analyses. The stress-tolerant yeast tolerated various chemical reagents and could grow with up to 600 g/L xylose for xylitol production. The yeast showed a broad carbohydrate utilization spectrum, with its greatest xylitol yield being in medium supplemented with xylose as the sole carbon source due to catabolic repression. Batch fermentation for xylitol production using xylose solution prepared from acidic EPFBF pretreatment wastewater showed lower xylitol yield compared to the culture supplemented with pure xylose. Nevertheless, XR containing the Ser279 residue in *C. tropicalis* YHJ1 allowed for more effective xylitol conversion compared to orthologous *Candida* enzymes containing Leu279 or Asn279. This residue might influence the tight interaction with the cofactor NADPH, as shown in the homologous protein simulation and enzymatic analysis. This study describes a novel stress-tolerant yeast strain and its XR, which can be used for xylitol production from a sugar solution based on biomass pretreatment wastewater.

## Data Availability

The raw data supporting the conclusions of this manuscript will be made available by the authors, without undue reservation, to any qualified researcher.

## Author Contributions

SK conceived and designed the experiment and performed the experiment. SK and BS analyzed data. JL performed the computational homologous protein modeling. All authors have wrote the manuscript, read, and approved the final manuscript.

### Conflict of Interest Statement

The authors declare that the research was conducted in the absence of any commercial or financial relationships that could be construed as a potential conflict of interest.

## References

[B1] Abdolali A. Guo W. S. Ngo H. H. Chen S. S. Nguyen N. C. Tung K. L. (2014). Typical lignocellulosic wastes and by-products for biosorption process in water and wastewater treatment: a critical review. Bioresour. Technol. 160, 57–66. 10.1016/j.biortech.2013.12.037 24405653

[B2] Alvira P. Tomás-Pejó E. Ballesteros M. Negro M. J. (2010). Pretreatment technologies for an efficient bioethanol production process based on enzymatic hydrolysis: a review. Bioresour. Technol. 101, 4851–4861. 10.1016/j.biortech.2009.11.093 20042329

[B3] Brethauer S. Studer M. H. (2015). Biochemical conversion processes of lignocellulosic biomass to fuels and chemicals - a review. Chimia 69, 572–581. 10.2533/chimia.2015.572 26598400

[B4] Brooks B. R. Brooks C. L. III. Mackerell A. D. Jr. Nilsson L. Petrella R. J. Roux B.. (2009). CHARMM: the biomolecular simulation program. J. Comput. Chem. 30, 1545–1614. 10.1002/jcc.21287 19444816 PMC2810661

[B5] Caicedo M. Barros J. Ordás B. (2016). Redefining agricultural residues as bioenergy feedstocks. Materials 9:E635. 10.3390/ma9080635 28773750 PMC5509081

[B6] Capolupo L. Faraco V. (2016). Green methods of lignocellulose pretreatment for biorefinery development. Appl. Microbiol. Biotechnol. 100, 9451–9467. 10.1007/s00253-016-7884-y 27714444 PMC5071362

[B7] Chen V. B. Arendall W. B. I. I.I. Headd J. J. Keedy D. A. Immormino R. M. Kapral G. J. (2010). MolProbity: all-atom structure validation for macromolecular crystallography. Acta Crystallogr. D66, 12–21. 10.1107/S0907444909042073 PMC2803126 20057044

[B8] Goli J. K. Panda S. H. Linga V. R. (2012). “Molecular mechanism of D-xylitol production in yeasts: focus on molecular transportation, catabolic sensing and stress response,” in D-xylitol, eds da Silva S. S. Chandel A. K. (Berlin: Springer-Verlag), 85–107. 10.1007/978-3-642-31887-0_4

[B9] Homann O. R. Dea J. Noble S. M. Johnson A. D. (2009). A phenotypic profile of the *Candida albicans* regulatory network. PLoS Genet. 5:e1000783. 10.1371/journal.pgen.1000783 20041210 PMC2790342

[B10] Humbird D. Davis R. Tao L. Kinchin C. Hsu D. Aden A. (2011). Process Design and Economics for Biochemical Conversion of Lignocellulosic Biomass to Ethanol. Dilute-Acid Pretreatment and Enzymatic Hydrolysis of Corn Stover, NREL/TP-5100-47764. Available online at: https://www.nrel.gov/docs/fy11osti/47764.pdf. 10.2172/1013269 (accessed May, 2011)

[B11] Islam M. N. Khalil M. I. Islam M. A. Gan S. H. (2014). Toxic compounds in honey. J. Appl. Toxicol. 34, 733–742. 10.1002/jat.2952 24214851

[B12] Jönsson L. J. Martín C. (2016). Pretreatment of lignocellulose: formation of inhibitory by-products and strategies for minimizing their effects. Bioresour. Technol. 199, 103–112. 10.1016/j.biortech.2015.10.009 26482946

[B13] Kim S. (2018a). A novel core 1 O-linked glycan-specific binding lectin from the fruiting body of *Hericium erinaceus*. Int. J. Biol. Macromol. 107, 1528–1537. 10.1016/j.ijbiomac.2017.10.018 28988842

[B14] Kim S. (2018b). Evaluation of alkali-pretreated soybean straw for lignocellulosic bioethanol production. Int. J. Polym. Sci. 2018:5241748 10.1155/2018/5241748

[B15] Kim S. (2018c). Enhancing bioethanol productivity using alkali-pretreated empty palm fruit bunch fiber hydrolysate. Biomed Res. Int. 2018:5272935. 10.1155/2018/5272935 30255095 PMC6145314

[B16] Kim S. (2019). Xylitol production from the byproducts generated in sequential acid-/alkali-pretreatment of empty palm fruit bunch fiber by an adapted *Candida tropicalis*. Front. Energ. Res. 10.1016/j.biortech.2012.01.036 22306078

[B17] Kim S. Kim C. H. (2012). Production of cellulose enzymes during the solid-state fermentation of empty palm fruit bunch fiber. Bioproc. Biosyst. Eng. 35, 61–67. 10.1007/s00449-011-0595-y 22052232

[B18] Kim S. Kim C. H. (2013). Bioethanol production using the sequential acid/alkali-pretreated empty palm fruit bunch fiber. Renew. Energ. 54, 150–155. 10.1016/j.renene.2012.08.032

[B19] Kim S. Kim C. H. (2014). Evaluation of whole Jerusalem artichoke (Helianthus tuberosus L.) for consolidated bioprocessing ethanol production. Renew. Energ. 65, 83–91. 10.1016/j.renene.2013.07.025

[B20] Kim S. Park J. M. Kim C. H. (2013). Ethanol production using whole plant biomass of Jerusalem artichoke by *Kluyveromycs marxianus* CBS1555. Appl. Biochem. Biotechnol. 169, 1531–1545. 10.1007/s12010-013-0094-5 23322254

[B21] Kim S. Park J. M. Seo J. W. Kim C. H. (2012a). Sequential acid-/alkali-pretreatment of empty palm fruit bunch fiber. Bioresour. Technol. 109, 229–233. 10.1016/j.biortech.2012.01.036 22306078

[B22] Kim T. R. Oh S. Yang J. S. Lee S. Shin S. Lee J. (2012b). A simplified homology-model builder toward highly protein-like structures: an inspection of restraining potentials. J. Comput. Chem. 33, 1927–1935. 10.1002/jcc.23024 22648914

[B23] Kratzer R. Leitgeb S. Wilson D. K. Nidetzky B. (2006). Probing the substrate binding site of *Candida tenuis* xylose reductase (AKR2B5) with site-directed mutagenesis. Biochem. J. 393, 51–58. 10.1042/BJ20050831 16336198 PMC1383663

[B24] Laskowski R. A. MacArthur M. W. Moss D. S. Thornton J. M. (1993). PROCHECK- a program to check the stereochemical quality of protein structures. J. App. Cryst. 26, 283–291. 10.1107/S0021889892009944

[B25] Lima L. H. das Graças de Almeida Felipe M. Vitolo M. Torres F. A. (2004). Effect of acetic acid present in bagasse hydrolysate on the activities of xylose reductase and xylitol dehydrogenase in *Candida guilliermondii*. Appl. Microbiol. Biotechnol. 65, 734–738. 10.1007/s00253-004-1612-8 15107950

[B26] Liu W. Wang J. Richard T. L. Hartley D. S. Spatari S. Volk T. A. (2017). Economic and life cycle assessments of biomass utilization for bioenergy products. Biofuel. Bioprod. Bior. 11, 633–647. 10.1002/bbb.1770

[B27] Liu Z. L. (2011). Molecular mechanisms of yeast tolerance and *in situ* detoxification of lignocellulose hydrolysates. Appl. Microbiol. Biotechnol. 90, 809–825. 10.1007/s00253-011-3167-9 21380517

[B28] Melin K. Hurme M. (2011). Lignocellulosic biorefinery economic evaluation. Cellulose Chem. Technol. 45, 443–454. Available online at: https://www.cellulosechemtechnol.ro/pdf/CCT45,7-8(2011)/p.443-454.pdf

[B29] Modig T. Lidén G. Taherzadeh M. J. (2002). Inhibition effects of furfural on alcohol dehydrogenase, aldehyde dehydrogenase and pyruvate dehydrogenase. Biochem. J. 363, 769–776. 10.1042/bj3630769 11964178 PMC1222530

[B30] Mpabanga T. P. Chandel A. K. da Silva S. S. Singh O. V. (2012). “Detoxification strategies applied to lignocellulosic hydrolysates for improved xylitol production,” in D-xylitol, eds da Silva S. S. Chandel A. K. (Berlin: Springer-Verlag), 63–82. 10.1007/978-3-642-31887-0_3

[B31] Palmqvist E. Hahn-Hägerdal B. (2000). Fermentation of lignocellulosic hydrolysates. II: inhibitors and mechanisms of inhibition. Bioresour. Technol. 74, 25–33. 10.1016/S0960-8524(99)00161-3

[B32] Peltroche-Llacsahuanga H. Schmidt S. Seibold M. Lütticken R. Haase G. (2000). Differentiation between *Candida dubliniensis* and *Candida albicans* by fatty acid methyl ester analysis using gas-liquid chromatography. J. Clin. Microbiol. 38, 3696–3704. 10.1016/S0732-8893(00)00205-4 11015386 PMC87459

[B33] Porman A. M. Alby K. Hirakawa M. P. Bennett R. J. (2011). Discovery of a phenotypic switch regulating sexual mating in the opportunistic fungal pathogen *Candida tropicalis*. Proc. Natl. Acad. Sci. U.S.A. 108, 21158–21163. 10.1073/pnas.1112076109 22158989 PMC3248515

[B34] Roncero C. Durán A. (1985). Effec of calcofluor white and congo red on fungal cell wall morphogenesis: *in vivo* activation of chitin polymerization. J. Bacteriol. 163, 1180–1185. 10.1128/jb.163.3.1180-1185.1985 PMC219256 3897187

[B35] Silveira M. H. Morais A. R. da Costa Lopes A. M. Olekszyszen D. N. Bogel-Łukasik R. Andreaus J.. (2015). Current pretreatment technologies for the development of cellulosic ethanol and biorefineries. ChemSusChem 8, 3366–3390. 10.1002/cssc.201500282 26365899

[B36] Thompson O. A. Hawkins G. M. Gorsich S. W. Doran-Peterson J. (2016). Phenotypic characterization and comparative transcriptomics of evolved *Saccharomyces cerevisiae* strains with improved tolerance to lignocellulosic derived inhibitors. Biotechnol. Biofuels 9:200. 10.1186/s13068-016-0614-y 27679668 PMC5029107

[B37] Vriend G. (1990). WHAT IF: a molecular modeling and drug design program. J. Mol. Graph. 8, 52–56. 10.1016/0263-7855(90)80070-V 2268628

[B38] Wang L. Wu D. Tang P. Yuan Q. (2013). Effect of organic acids found in cottonseed hull hydrolysate on the xylitol fermentation by *Candida tropicalis*. Bioprocess Biosyst. Eng. 36, 1053–1061. 10.1007/s00449-012-0858-2 23138642

[B39] Wang S. Li H. Fan X. Zhang J. Tang P. Yuan Q. (2015). Metabolic responses in *Candida tropicalis* to complex inhibitors during xylitol bioconversion. Fungal Genet. Biol. 82, 1–8. 10.1016/j.fgb.2015.04.022 26127015

[B40] Wang S. Sun X. Yuan Q. (2018). Strategies for enhancing microbial tolerance to inhibitors for biofuel production: a review. Bioresour. Technol. 258, 302–309. 10.1016/j.biortech.2018.03.064 29567023

[B41] Zhang J. Geng A. Yao C. Lu Y. Li Q. (2012a). Xylitol production from D-xylose and horticultural waste hemicellulosic hydrolysate by a new isolate of *Candida athensensis* SB18. Bioresour. Technol. 105, 134–141. 10.1016/j.biortech.2011.11.119 22196071

[B42] Zhang J. Geng A. Yao C. Lu Y. Li Q. (2012b). Effects of lignin-derived phenolic compounds on xylitol production and key enzyme activities by a xylose utilizing yeast *Candida athensensis* SB18. Bioresour. Technol. 121, 369–378. 10.1016/j.biortech.2012.07.020 22864173

[B43] Zhang Y. Skolnick I. (2005). TM-align: a protein structure alignment algorithm based on the TM-score. Nucleic Acids Res. 33, 2302–2309. 10.1093/nar/gki524 15849316 PMC1084323

[B44] Zirbes L. Nguyen B. K. de Graaf D. C. De Meulenaer B. Reybroeck W. Haubruge E.. (2013). Hydroxymethylfurfural: a possible emergent cause of honey bee mortality. J. Agric. Food. Chem. 61, 11865–11870. 10.1021/jf403280n 24127696

