# Supplementary material for: Isolation and Characterization of the Stress-Tolerant Candida tropicalis YHJ1 and Evaluation of Its Xylose Reductase for Xylitol Production From Acid Pre-treatment Wastewater
Source: Front Bioeng Biotechnol. 2019 Jul 2;7:138. doi: 10.3389/fbioe.2019.00138 (PMC6626919; doi:10.3389/fbioe.2019.00138)
Supplement: Supplementary file 1 [file Data_Sheet_1.docx]

**Supplementary data 1.**

**Figure Legends**

**Figure S1. Phylogenetic trees based on sequences of the D1/D2 region (A) and ITS1/ITS4 region (B) of the rDNA gene of the yeast used in this study, showing the position of *Candida tropicalis* YHJ1 with respect to closely related species.** The evolutionary history was inferred using the neighbor-Joining method [1]. The percentage of replicate trees in which the associated taxa clustered together in the bootstrap test (50 replicates) is shown next to each branch [2]. The tree is drawn to scale, with branch lengths in the same units as the evolutionary distances used to infer the phylogenetic trees. The evolutionary distances were computed using the maximum composite likelihood method [3] and are in units of number of base substitutions per site. Evolutionary analyses were conducted using MEGA7 [4].

**Figure S2**. **Morphological analysis of the isolated yeast strain.** (A) Scanning electron microscopy (SEM) images of *Candida tropicalis* YHJ1 cultivated in YPD medium. The yeast was fixed after 48 h of cultivation at 30 °C. White box (left panel) indicates zoom in view of the yeast (right panel) (B) Comparison of the colony color of *Candida tropicalis* and *Candida albicans* strains on CHROMagar™ Candida agar plate. Metallic blue colonies of *C. tropicalis* YHJ1 or CBS94 and deep green colonies of *C. albicans* MYA-682 or 2876 were observed on the indicator plate. The yeast strains were grown for 48 hours on CHROMagar Candida at 30°C.

**Figure S3. Expression of recombinant *C. tropicalis* YHJ1 XR (wild-type XR) and its mutant proteins (XR_S279L and XR_S279N) in *Escherichia coli*.** (A) Tricine-PAGE (left) and western blot (right) analysis of the recombinant XR (wild-type protein, WT) and its mutant proteins (S279L and S279N). The proteins expressed in the cell lysates were detected using anti-His antibody as a primary antibody in western blot analysis. The -/+ headers indicate the presence of the inducer IPTG in the culture broth. (B) Tricine-PAGE analysis of the recombinant XR and its mutant proteins purified using chromatography techniques described in the materials and methods section. Lane 1, cell lysate; lane 2, pass-through fraction; lane 3, purified proteins in the affinity chromatography. The arrows indicate the recombinant XR and its mutant proteins.

**Figure S4**. **Morphological analysis of *C. tropicalis* YHJ1 cultivated in culture medium containing a high concentration of xylose.** SEM of *Candida tropicalis* YHJ1 cultivated in semi-defined medium containing various xylose concentrations ranging from 100 to 600 g/L. The cultivated yeasts were harvested and then fixed for SEM analysis.

**Figure S1.**

**(A)**

**
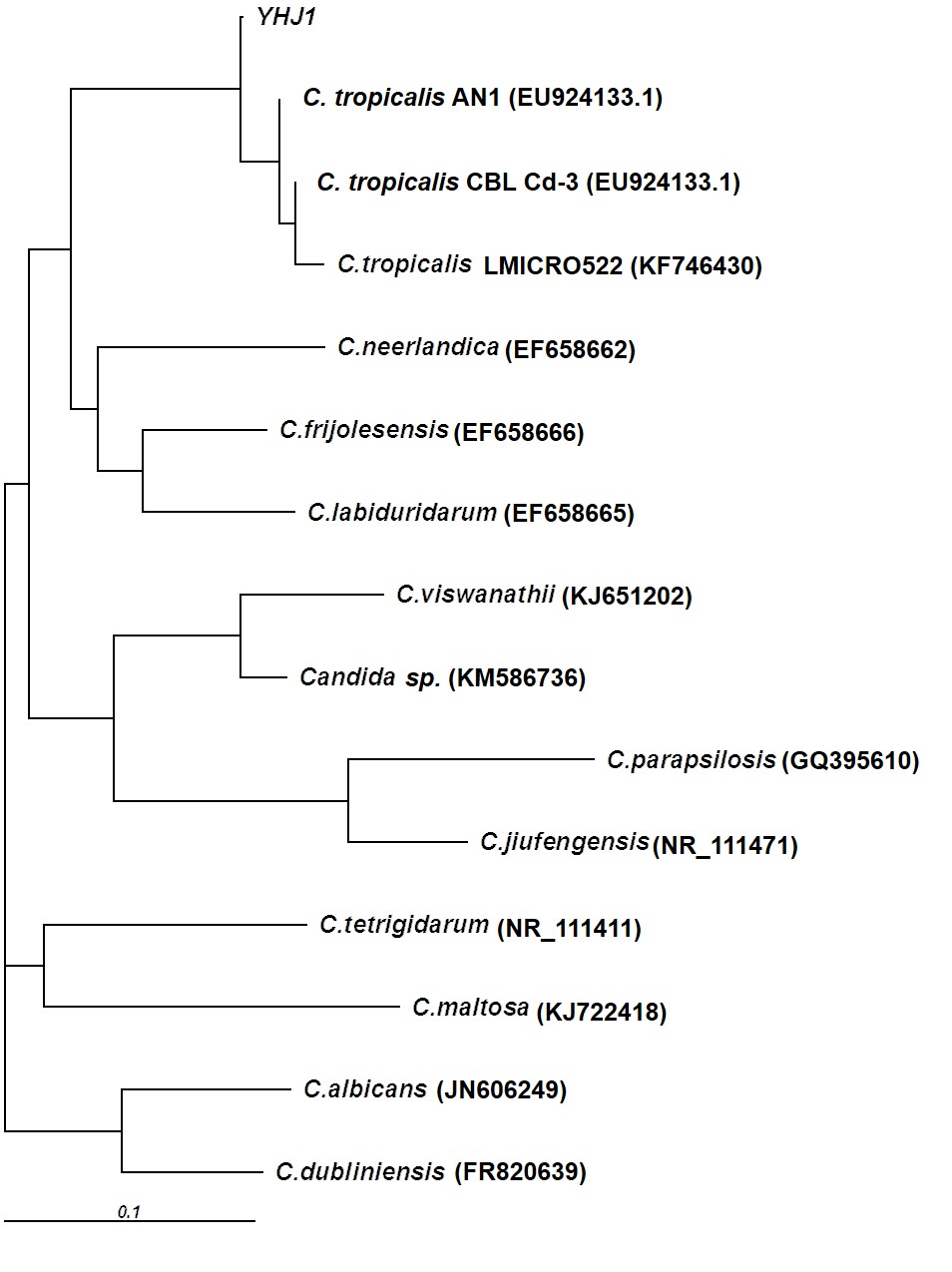
**

*(Continue)*

**(B)**

**
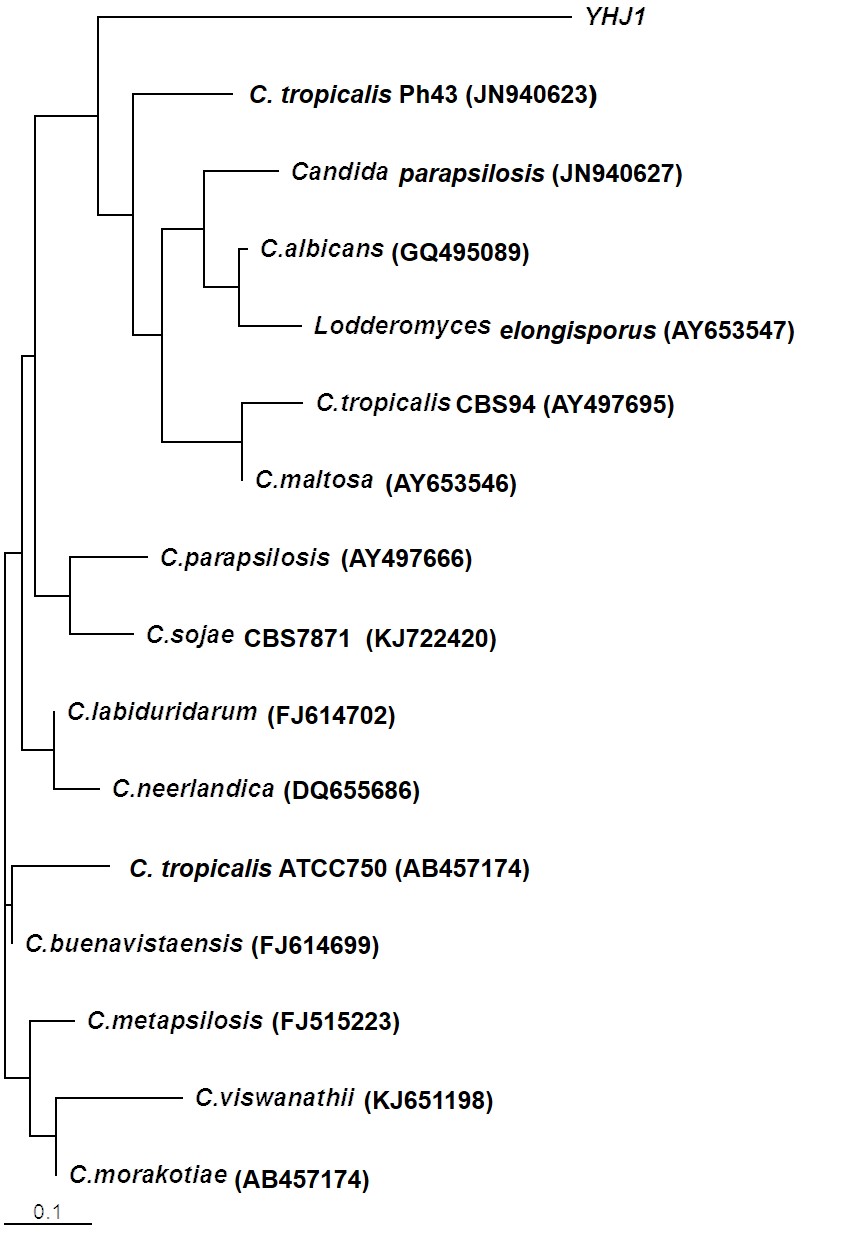
**

**Figure S2.**

**
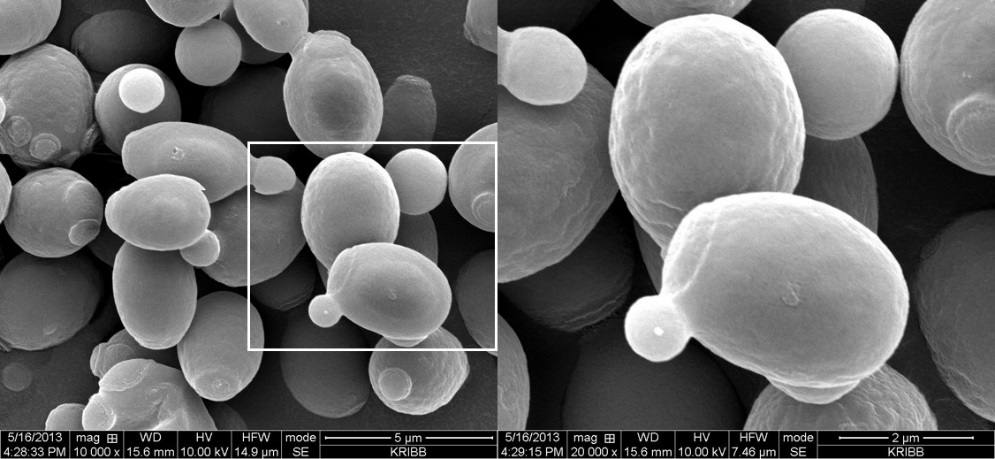
(A)**

**
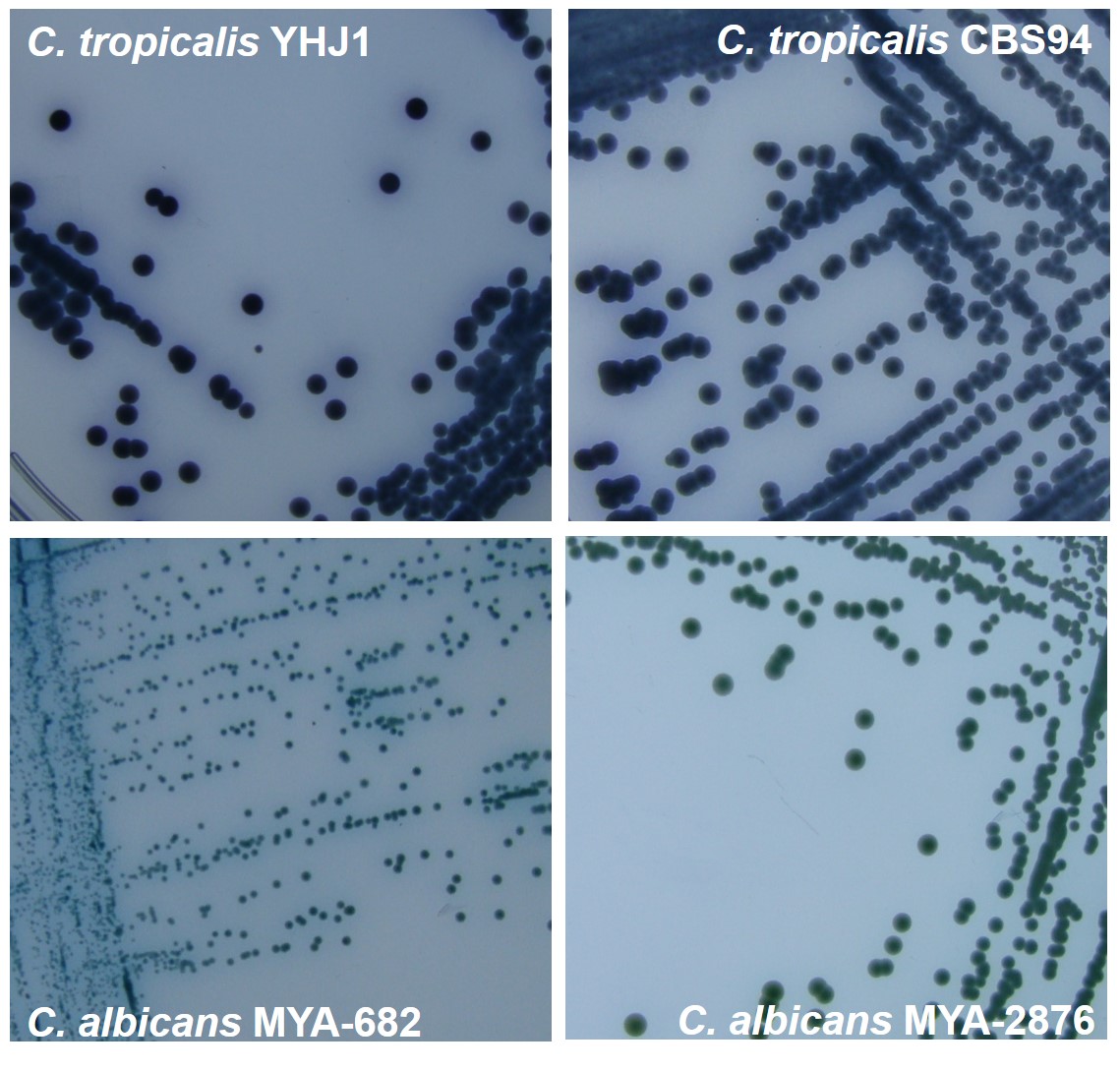
(B)**

**Figure S3.**

**
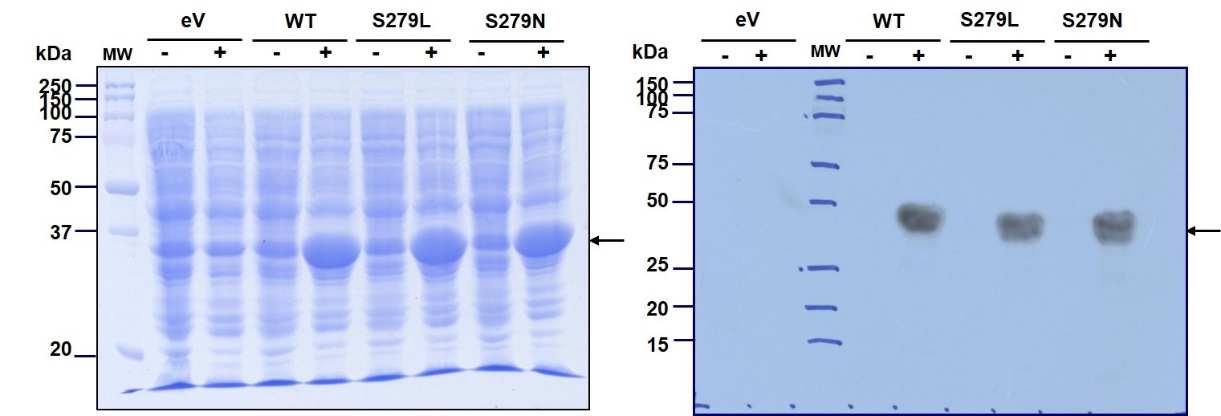
 (A)**

**
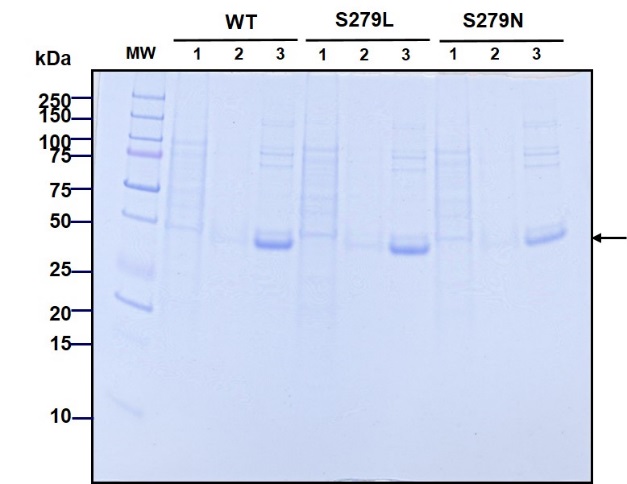
(B)**

**
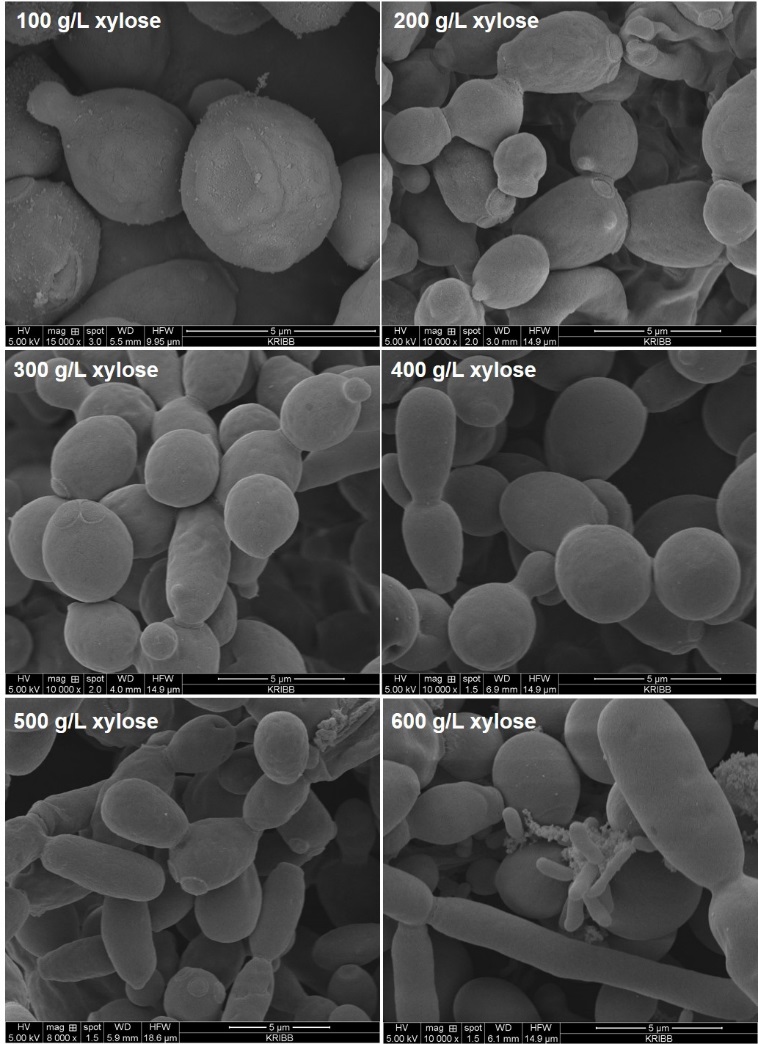
Figure S4.**

**References**

1. N. Saitou, M. Nei, The neighbor-joining method: A new method for reconstructing phylogenetic trees, Mol. Biol. Evol. 4 (1987) 406-425.
2. J. Felsenstein, Confidence limits on phylogenies: An approach using the bootstrap, Evolution 39 (1985) 783-791.
3. K. Tamura, M, Nei, S. Kumar, Prospects for inferring very large phylogenies by using the neighbor-joining method, Proc. Natl. Acad. Sci. (USA) 101 (2004) 11030-11035.
4. S. Kumar, G. Stecher, K. Tamura, MEGA7: Molecular Evolutionary Genetics Analysis version 7.0 for bigger datasets. Mol. Biol. Evol. 33 (2016) 1870-1874.
