# Supplementary material for: Isolation and Characterization of the Stress-Tolerant Candida tropicalis YHJ1 and Evaluation of Its Xylose Reductase for Xylitol Production From Acid Pre-treatment Wastewater
Source: Front Bioeng Biotechnol. 2019 Jul 2;7:138. doi: 10.3389/fbioe.2019.00138 (PMC6626919; doi:10.3389/fbioe.2019.00138)
Supplement: Supplementary file 2 [file Data_Sheet_2.docx]

**Supplementary data 2.**

**Table S1. Carbohydrate utilization analysis**

| Carbon source | Utilization | |
| --- | --- | --- |
|  | *C. tropicalis* YHJ1 | *C. tropicalis* CBS92^1)^ |
| Hexose |  |  |
| D-Fructose | +++++ | n.d.^2)^ |
| D-Glucose | +++++ | + |
| D-Galactose | +++++ | + |
| D-Mannose | +++++ | n.d.^2)^ |
| L-Rhamnose | - | - |
|  |  |  |
| Pentose |  |  |
| L-Arabinose | (+)^3)^ | - |
| D-Arabinose | - | - |
| D-Xylose | +++++ | + |
| D-Ribose | - | - |
|  |  |  |
| Disaccharide |  |  |
| Lactose | - | - |
| Cellobiose | +++^4)^ | + |
| Sucrose | +++++ | + |
| Maltose | +++++ | + |
| Melibiose | - | - |
| Trehalose | ++++ | +^4)^ |
|  |  |  |
| Trisaccharide |  |  |
| D-Raffinose | ++++ | - |
|  |  |  |
| Polysaccharide |  |  |
| Starch | (+)^3)^ | + |
| Carboxymethylcellulose | - | n.d.^2)^ |
| Avicel | - | n.d.^2)^ |
| Xylan oat spelt | (+)^3)^ | n.d.^2)^ |
| Xylan birch wood | - | n.d.^2)^ |
| Xylan beech wood | - | n.d.^2)^ |
| Inulin | (+)^3)^ | - |
|  |  |  |
| Sugar alcohol |  |  |
| Glycerol | +++^4)^ | + |
| Sorbitol | +++++ | n.d.^2)^ |
| Mannitol | +++++ | + |
| Xylitol | +++^4)^ | - |
|  |  |  |
| Sugar acid |  |  |
| D-Gluconic acid | ++++ | + |
| D-Xylonic acid | (+)^3)^ | - |
| D-Glucuronic acid | - | - |
| D-Galacturonic acid | - | - |
|  |  |  |
| Alcohol |  |  |
| Methanol | - | - |
| Ethanol | +++++ | + |
|  |  |  |
| Sugar amine |  |  |
| N-Acetylglucosamine | +++++ | n.d.^2)^ |

1) Cell growth data were obtained from the Centraalbureau voor Schimmelcultures (CBS) Fungal Biodiversity Centre - an Institute of the Royal Netherlands Academy of Arts and Sciences (KNAW). http://www.cbs.knaw.nl/collections/BioloMICS.aspx?Table=Yeasts%20species&Name=Candida%20tropicalis&ExactMatch=T

2) n.d. not determined

3) Cell growth was less than 0.01 OD 600 nm

4) Cell growth was very slow.
